# Creative ideas generation and personality: evidence from process communication model

**DOI:** 10.3389/fpsyg.2024.1403714

**Published:** 2024-06-13

**Authors:** Sixtine Lefebvre, Anaëlle Camarda

**Affiliations:** ^1^Kahler Communication France, Croisy-sur-Eure, France; ^2^Paris-Cité University, LaPEA, Boulogne Billancourt, France; ^3^Institut Supérieur Maria Montessori, Paris, France

**Keywords:** creativity, personality, process communication model, fluency, flexibility

## Abstract

The present study investigated the relation between personality and ideas generation abilities. Ideas generation was assessed by the “egg task” in which participants had to generate as many solutions as possible to design ways to drop a hen’s egg from a height of 10 m so that it does not break. The 102 participants were also presented with the standard Process Communication Model (PCM) questionnaire. Results suggest that idea generation varied according to PCM Base Type of participants. Even if five out of six Base Types (Thinker, Persister, Harmonizer, Promoter and Rebel) presented similar fluency and categorical flexibility, Imaginer Base presented higher scores than other Base Types. These results, discussed according to cognitive control abilities, reinforce the view that PCM can highlight an individual’s creative performance considering interindividual differences.

## 1 Introduction

Creativity is a human skill that has fascinated researchers for decades (Runco and Jaeger, 2012; Benedek et al., 2014; Cassotti et al., 2016; Camarda et al., 2018). Today, its understanding is important since it has been referenced as one of the four key skills of the 21st century (Thornhill-Miller et al., 2023). It is therefore essential to determine the factors that impact it. A well-documented literature showed that specific personality traits such as Openness to experience (i.e., the extent to which participants are curious, open-minded, and imaginative) or Extraversion (i.e., energy, positive affect, sociability, enthusiasm, novelty seeking, dominance, self-confidence; Costa and McCrae, 1992) are positively linked to creative performances (Karwowski and Lebuda, 2016; Kaspi-Baruch, 2019; Grajzel et al., 2023). More precisely, they seem to be associated with the generative aspect of the creative process, the divergent thinking ability (Fürst et al., 2016), that is the ability to find numerous different original solutions to a given problem (McCrae, 1987; Karwowski and Lebuda, 2016; Kaspi-Baruch, 2019). However, most of these studies are based on the emblematic Big Five model (Costa and McCrae, 1992), whereas the link between creative performance and other personality models widely used in recruiting creative people and building creative teams in industries has not been studied. In this context, the present study aims to investigate for the first time whether there is a relation between an individual’s creativity and personality characteristics according to Process Communication Model (PCM; Kahler, 2008), often used in the field.

PCM was created in the 1970s and has gained visibility thanks to its intensive use within NASA, during the selection and training of astronauts (see, e.g., Kahler, 2008, 2013; McGuire, 2022). Today, 5,000 trainers and coaches are accredited to use it worldwide, across 54 countries and 24 different languages. In France, since 2012, 201 companies in various sectors (health and social support, education, banking, etc.) have received in-house training based on the use of PCM (Kahler Communication France; Kahler, 2013).

In this model, the Personality Structure is represented by the metaphor of a Condominium compound by 6 Floors (Kahler, 2008). The 1st Floor is the Base, the most developed Personality Type from birth, the one with which people prefer to communicate with and where Character Strengths are strongest. Once this Base is set, it remains stable over time (Stansbury, 1990). The other Floors are layered above the Base. Each of us has a Personality Structure made up of the six Personality Types in a different order: Thinker, Persister, Harmonizer, Rebel, Imaginer, Promoter. We have a Personality Type on each Floor. We exhibit the characteristics of all of them. Each Type has its own Character Strengths (Kahler, 2008, 2013): When we activate the Thinker Floor we are responsible, logical, and organized; at the Persister Floor we are dedicated, observant and conscientious; at the Harmonizer Floor we are compassionate, sensitive and warm; at the Rebel Floor we are spontaneous and playful; at the Imaginer Floor we are imaginative, reflective and calm; and at the Promoter Floor we are adaptative, charming and persuasive.

There are six Perceptions by which we experience, interpret, and respond to our environment and others. The most accessible one is the Base. Everyone can perceive the world in six different ways: through Perception of Thoughts at the Thinker Floor, Opinions at the Persister Floor, Emotions at the Harmonizer Floor, Inaction (reflections) at the Imaginer Floor, Reactions (likes/dislikes) at the Rebel floor and Actions at the Promoter Floor.

Given this model, each of the Base Type may influence creative ideas generation, especially the Imaginer Base (Kahler, 2008, 2013). In our previous study we found a relation between Base Type and visuospatial processing (Lefebvre and Beaucousin, 2023). Even when objectively presented with similar visual stimuli, individual responses differed according to the participants’ Base Type. Although four out of six Base Types (Thinker, Persister, Harmonizer and Promoter) showed classic way of visuo-spatial processing (i.e., correct detection of a visual target is influenced by the number of visual distractors on the screen), Rebel and Imaginer Base Types showed different processing of visual distractors than other Base Types. In particular, Rebel Base participants were highly sensitive to the number of visual distractors presented during the task, whereas Imaginer Base participants were not. Therefore, it seems quite conceivable that Rebel Base and Imaginer Base participants could also differ in other cognitive processes, such as ideas generation.

As discussed above, one of the key processes in someone’s ability to generate creative ideas is his/her divergent thinking ability, i.e., the ability to generate many divergent alternatives in a number of different ways of solving a problem (Acar and Runco, 2019). To succeed in a creative task, one has to be capable of ideational fluency (i.e., generating many ideas), to explore many different paths of solutions (i.e., flexibility) and generate new and rare ideas (i.e., originality). Various measures of originality have been proposed in the literature, one of which is to differentiate between ideas that are more widely used and those that are less so. Agogué et al. (2014) demonstrated that when participants were asked to generate numerous creative solutions to ensure that an egg dropped from a height of 10 m did not break, 80% of adult’s responses fell into just three of the ten solution categories. These last consist of damping the shock (i.e., using a mattress), slowing the fall (i.e., using a parachute) and protecting the egg (i.e., creating a box around the egg). According to the triadic model of creativity (Cassotti et al., 2016), this fixation effect comes from the activation of categories that are easily accessible because the associated knowledge are automatically and intuitively using a first system of thought (system 1: automatic, effortless and intuitive). To be creative, one needs to overcome these fixation effects and engage a slower and more analytical system of thought (system 2) that would allow us to explore the other seven categories deemed more creative (e.g., training an eagle to catch the egg, see Agogué et al., 2014). Recent studies have supported this dual model by highlighting that ideas generated by fixation and expansion represent two distinct cognitive processes of ideational fluency (Camarda et al., 2021; Kruse et al., 2023). One is the automatic generation of ideas, which is considered uncreative, and the other is considered original because it is rarely given. Thus, it is considered that the more ideas an individual is able to generate, the more creative he or she will be judged to be.

The scientific literature already highlighted that creative persons score higher on openness to experience and extraversion scales. Indeed, well-documented literature shows that specific personality traits such as Openness to experience (i.e., the extent to which participants are curious, open-minded and imaginative) or Extraversion (i.e., energy, positive affect, sociability, enthusiasm, novelty seeking, dominance, self-confidence; Costa and McCrae, 1992) are positively linked to creative performances (Karwowski and Lebuda, 2016; Kaspi-Baruch, 2019; Grajzel et al., 2023). More precisely, they seem to be associated with the generative aspect of the creative process, the divergent thinking ability (Fürst et al., 2016), that is the ability to find numerous different original solutions to a given problem (McCrae, 1987; Karwowski and Lebuda, 2016; Kaspi-Baruch, 2019). However, most of these studies are based on the emblematic Big Five model (Costa and McCrae, 1992; Sung and Choi, 2009). Thus, the present work aimed to further investigate the relation between interindividual Personality Structure and the ability to generate creative ideas during the egg task presented above.

## 2 Materials and methods

### 2.1 Participants

This study included 102 participants (53 women and 49 men, *M* = 40.1 years, *SD* = 8.8). All participants provided written informed consent. An *a-priori* power analysis using G*Power 3.1 (Faul et al., 2007) was conducted with a mixed 6 × 2 design with one between-subject factor of group (participant’s Base Type: Thinker Base, Persister Base, Harmonizer Base, Rebel Base, Imaginer Base, Promoter Base) and two within-subject factors (which represents the analysis that will be carried out and requires the highest sample size) indicated that a sample size of 60 participants (10 per group) would be sufficient to detect a medium effect size (*f* = 0.25) with a power of 0.80 and an alpha of 0.05.

### 2.2 Experimental procedure

At home, the participants were asked to complete PCM questionnaire (Stansbury, 1990; Kahler, 2008), composed of 45 multiple-choice questions. For each question, six choices representing the six different personality characteristics were offered. The participants could select a maximum of 5 choices, and had to rank the answers in order of importance, from the 1st “most important” choice to the 5th “least important” choice. Participant’s Base Type was deduced from their responses to the questionnaire.

**Figure 1:**
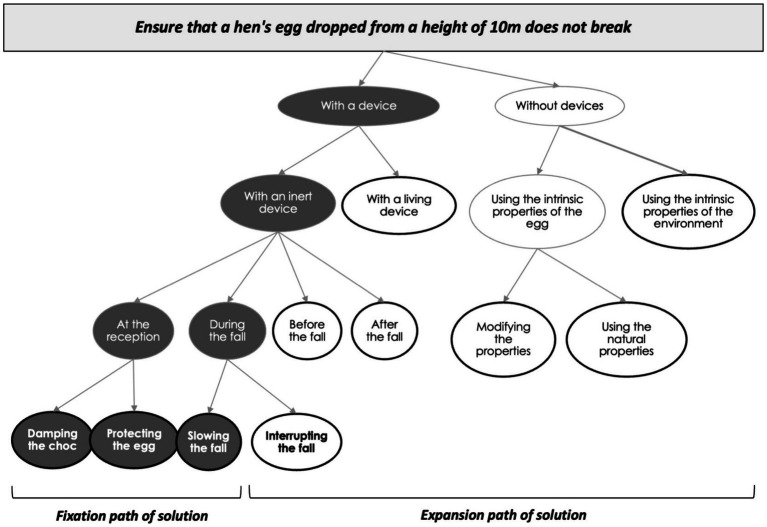
Representation of the fixation and expansion path of solution to the egg task used in the present study following the methodology provided by Agogué et al. (2014) and Camarda and Cassotti (2024).

Then, in a laboratory environment, participants were asked to solve the egg task. They had 10 min to generate as many original solutions as possible to the following problem: “*You are a designer, and you have to find as many original solutions as possible to the following problem: ensure that a hen’s egg dropped from a height of 10 m does not break.”* The task was analyzed according to the previously published procedure (Agogué et al., 2014; Camarda et al., 2021; see Camarda and Cassotti (2024) to access to material for analysis). Two trained experimenters assigned each response to one of 60 solution sub-categories. Each of these was assigned to one of the 10 meta-categories of the task, 3 of which represented the solution fixation path (i.e., damping the shock, using a mattress; Protecting the egg, using a cotton around the egg to protect it; Slowing the fall, hanging the egg to a parachute), and 7 of them representing the expansive path of solution (i.e., Interrupting the fall, by using a net a few centimeters below the launch; Acting before the fall, for instance by dropping the egg from a height of 11 m; Acting after the fall, for instance by replacing the broken egg with a new one; Using a living device, for instance by training an eagle, to catch the egg; Modifying the properties of the egg, for instance by freezing the egg before dropping it; Using the natural properties of the egg, for instance by dropping the egg on its strongest axis; Using the properties of the environment, for instance by dropping the egg when there is no gravity; see Figure 1). For each participant, different scores were calculated: the fluency score (i.e., the number of ideas generated), the flexibility score (i.e., the number of sub-categories explored), the fixation score (i.e., the number of ideas generated within the solution fixation path), and the expansion score (i.e., the number of ideas generated outside the solution fixation path). Note that the fluency score is the sum of the number of fixations and the number of expansions.

## 3 Results

Among all participants, PCM questionnaire indicated that 17 participants had a Thinker Base, 15 a Persister Base, 23 a Harmonizer Base, 17 a Rebel Base, 15 an Imaginer Base and 15 a Promoter Base.

**Figure 2:**
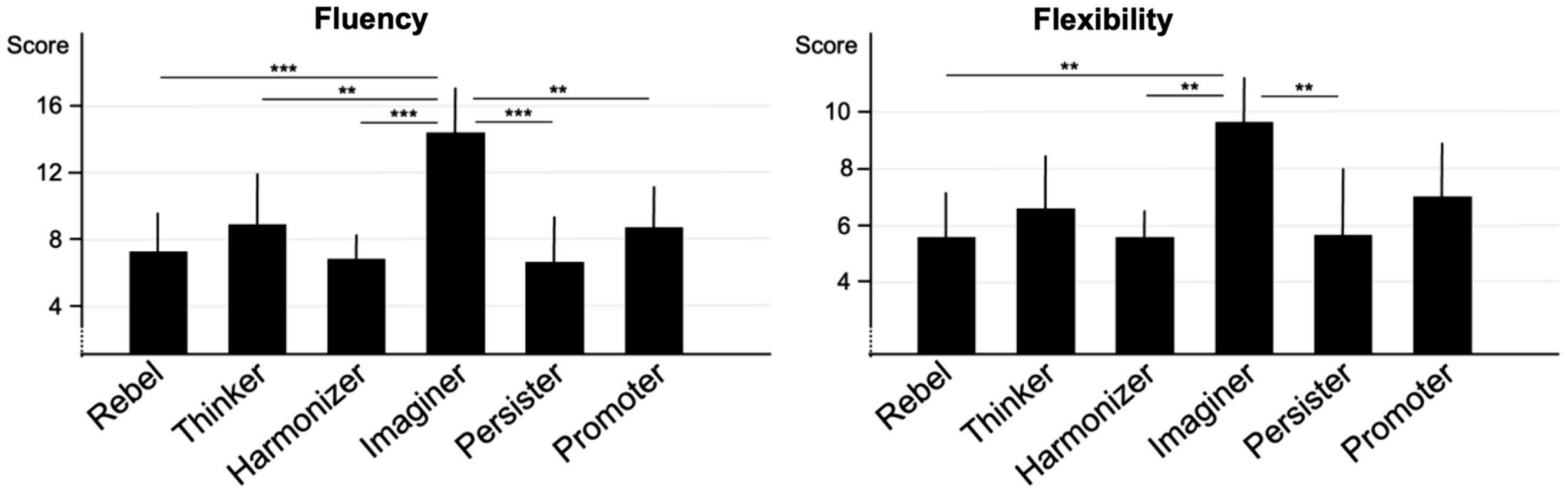
Results for fluency, and flexibility scores according to Base Types. *** *p* < 0.001, ** *p* < 0.01, * *p* < 0.05.

To examine whether the number of proposed solutions varied according to the participants’ Base, we performed a one-way analysis of variance (ANOVA) for the fluency score, with Base Type as a between-subjects factor, and *post hoc* comparisons using holm Bonferroni corrections. The results show a significant effect of Base Type, *F* (5, 96) = 5.96, *p* < 0.001, η_p_^2^ = 0.237, characterized by higher scores for Imaginer Base than for other Base Types (Imaginer: *M =* 14.5, *SD* = 4.8 vs. Rebel: *M =* 7.29, *SD* = 4.44, *p* < 0.001; vs. Thinker: *M =* 8.82, *SD =* 6.3, *p* = 0.01; vs. Harmonizer: *M =* 6.9, *SD =* 3.4, *p* < 0.001; vs. Persister: *M =* 6.53, *SD =* 5.12, *p* < 0.001; vs. Promoter: *M =* 8.6, *SD =* 4.8 *p* = 0.01, see Figure 2). All other comparisons were not significant (all *p*s > 0.05).

The ANOVA examining the impact of Base Type on flexibility score revealed a significant effect, *F* (5, 96) = 3.72, *p* = 0.004, η_p_^2^ = 0.162, characterized by higher scores for Imaginer Base than Rebel Base (Imaginer: *M =* 9.67, *SD =* 2.84 vs. Rebel: *M =* 5.53, *SD =* 3.22, *p* = 0.008), Harmonizer Base (*M =* 5.56, *SD =* 2.39, *p* = 0.004) and Persister Base (*M =* 5.67, *SD =* 4.43, *p* = 0.02). Their results were similar to those of Thinker Base (*M =* 6.64, *SD =* 3.53, *p* = 0.17) and Promoter Base (*M =* 7.07, *SD =* 3.37, *p* = 0.49; see Figure 2). All comparisons between other Base Types were not significant (all *p*s > 0.05).

Finally, a repeated-measures ANOVA with Base Type as the between-subjects factor and responses type (Fixation and Expansion) as the within-subjects factor demonstrated a main effect of response type (*F* (1, 96) = 47.58, *p* < 0.001, η_p_^2^ = 0.331), revealing more fixation than expansion responses for each Base Type. (*F* (5, 96) = 5.96, *p* < 0.001, η_p_^2^ = 0.237; Imaginer: *M_Fixation_ =* 9.4, *SD_Fixation_ = 4.47*, *M_Expansion_ =* 5.13, *SD_Expansion_ =* 3.48; Rebel: *M_Fixation_ =* 4.23, *SD_Fixation_ =* 3.29, *M_Expansion_ = 3.06*, *SD_Expansion_ =* 2.25; Thinker: *M_Fixation_ =* 6.29, *SD_Fixation_ = 3.12*, *M_Expansion_ =* 2.52, *SD_Expansion_ =* 3.46; Harmonizer: *M_Fixation_ =* 5.00, *SD_Fixation_ =* 2.89, *M_Expansion_ =* 1.91, *SD_Expansion_ =* 1.76; Persister: *M_Fixation_ =* 4.07, *SD_Fixation_ =* 3.5, *M_Expansion_ =* 2.47, *SD_Expansion_ =* 2.26; Promoter: *M_Fixation_ =* 5.2, *SD_Fixation_ =* 3.19, *M_Expansion_ =* 3.4, *SD_Expansion_ =* 2.47;). Furthermore, there was no interaction between Base Type and response type, *F* (5, 96) = 1.80, *p* = 0.12, η_p_^2^ = 0.086.

It should be noted that additional multivariate analyses of variance (MANOVAs) including (1) Base Type, Flexibility and Fluency and (2) Base Type, Flexibility, Fixation and Expansion yielded similar results to the separated ANOVAs mentioned above (*F* (5, 96) = 3.14, *p* < 0.001 and *F* (5, 96) = 2.42, *p* = 0.003, respectively).

## 4 Discussion

In this study, we examined the relation between personality as measured by PCM questionnaire and the generation of creative ideas. The results showed that participants with Imaginer Base had higher fluency and flexibility scores than participants with other Base Types. However, they achieved similar results in terms of number of ideas generated within the fixation and the expansive paths of solution. Thus, although Imaginer Base participants are able to generate a greater number of ideas and explore a broader number of categories during a divergent thinking task, they are not able to specifically provide a creative exploration of the solution. Overall, our findings suggest that among Base Types, inter-individual peculiarities may affect how participants generate ideas during creative tasks.

Why did Imaginer Base participants perform better than other Base Types during ideas generation? In a recent study Lefebvre and Beaucousin (2023), Imaginer Base participants were more resistant to visual interference and exhibited a particular mode of visual processing compared with other Base Types participants; they were not sensitive to the distractors presented during a visuo-spatial task, whereas the reaction times of all other Base Types participants were slowed and altered by the presence of visual distractors during the task. In line with these findings, it seems conceivable that Imaginer Base participants are also less affected by the disruptive effects that impede idea generation during a creative task. Consequently, their ability to be less influenced by distractors could enable them to develop more solutions and more categories than other Base Types. The present results support the idea that, as with visual information, Imaginer Base participants may be more willing to process cognitive mechanisms than other Base Types to overcome fixation effect in order to generate new ideas. One might think that, according to this hypothesis, Imaginer Base participants would perform better in the expansion categories (and not in the fixation categories). This was not the case in the present results since Imaginer Base participants were more fluent in both expansion and fixation categories.

From a practical point of view, Imaginer Base people are known to be calm, imaginative, and reflective people (Kahler, 2008; Dufourneaud and Heffta, 2022). They perceive the world through Inaction (Reflections), which means they have to think, to reflect and they need time to muse. They feel good in a calm environment and their psychological need is solitude. They are visionaries and can imagine an infinite range of possibilities. Most of the time, if no one gives them clear direction in terms of work, they would remain silent and may say that they were not told what to do. According to the present results, promoting ideas generation by Imaginer Base Type could provide a significant benefit during creative situations. On a theoretical level in PCM, Imaginer Base people are withdrawn from the relationship, they need external stimulation from their environment to share what is on their minds and to take an active part in meetings. When recruiting participants we need to keep this information in mind, to prevent the Imaginer Base Type from being underrepresented in future scientific research. In the day-to-day business world, Imaginer Base people will be very useful in bringing different perspectives and innovative solutions to the table. Even if their ratio between the number of ideas generated in fixation and expansion seems similar to those of the other Base type, Imaginers Base are more likely to reach creative solutions than other participants. Indeed, the more one is able to generate a large number of solutions (i.e., fluency), the more chance we have of reaching creative ideas. Thus, it would be interesting to study the profiles of eminently creative persons, and check whether the Imagine floor would be their Base. In fact, video analyzes carried out by PCM experts, speculate that Einstein may have had an Imaginer Base thanks to which he created the theory of relativity. It would therefore be relevant to deepen this hypothesis of the relationship between the Basic Imagineer and creativity skills by carrying out a study among eminently creative individuals.

Some limitations should be noted. Even if the present study is the first to demonstrate a link between PCM and divergent thinking abilities, future studies should explore other features of cognitive functions related to Base Types and other personality models, in particular the Big 5 model (Costa and McCrae, 1992), to better understand how Personality Structure may affect cognitive processes (Grajzel et al., 2023). We investigated the bridge between participants’ profiles according to their Base in PCM and the hypothesis derived from the existing literature on the link between creative performance and the personality of individuals. Nevertheless, no study has examined whether PCM interacts with other personality models, particularly the Big 5. Therefore, future studies should replicate our results and, at the same time, investigate the link between PCM and the Big Five traits to provide empirical evidence regarding the relation between the two personality models and creative abilities. Furthermore, creativity is a complex process, which can be measured using different methods depending on the targeted mechanisms of interest (Camarda and Cassotti, 2024). Measures of divergent thinking are the most widely used in the creativity literature. In this sense, the task we used in our study seems relevant since it measures individual’s fluency, flexibility, and his/her ability to resist to generativity biases (fixation effects). However, divergent thinking can be measured using other tasks such as the emblematic Alternative Use of Object (Guilford, 1967) or the Torrance Test (Torrance, 1966). Their link with creative achievement has been highlighted on numerous occasions, notably in a recent meta-analysis based on 766 effect sizes (Said-Metwaly et al., 2022). Despite this, the literature has shown that an individual’s creative potential depends on their cognitive, conative and socio-affective skills, but also on the modalities of the task and its domain (Camarda and Lubart, 2023; Camarda and Cassotti, 2024). In addition, other forms of thinking have been strongly associated with creativity, such as convergent thinking (Cropley, 2006), and involve cognitive processes that are different from and complementary to those required for divergent thinking (e.g., selection processes). Thus, future studies should replicate the present results while proposing a broader creativity test battery.

In conclusion, the present study highlights that the Process Communication Model, widely used to describe the personality of individuals in the field although little present in the scientific literature, offers a relevant theoretical framework which should be further explored in future studies. Indeed, beyond a simple description of different personalities type, the proposed classification reveals different performances in generating creative ideas as predicted by the PCM: Imagineers benefit from a greater capacity to generate numerous ideas and a better exploration of the different categories of possible solutions. Thus, having an Imaginer Base appears to impact not only the way we perceive visual information (Lefebvre and Beaucousin, 2023), but also individual’s ability to generate multiple creative solutions to a problem.

## Data availability statement

The datasets presented in this study can be found in online repositories. The names of the repository/repositories and accession number(s) can be found at: https://osf.io/ywq34/?view_only=e8ebc2eeff404190a26b412cc1aa4adf.

## Ethics statement

Ethical approval was not required for the studies involving humans because in France, the law does not require going to the Ethics Committee if the studies are not invasive. Thus, we were not required to obtain the opinion of an ethics committee in the case of this study. The studies were conducted in accordance with the local legislation and institutional requirements. The participants provided their written informed consent to participate in this study.

## Author contributions

SL: Conceptualization, Formal analysis, Methodology, Writing – original draft, Writing – review & editing. AC: Conceptualization, Formal analysis, Methodology, Writing – original draft, Writing – review & editing.
